# Efficacy and Safety of the Extreme Lateral Interbody Fusion (XLIF) Technique in Spine Surgery: Meta-Analysis of 1409 Patients

**DOI:** 10.3390/jcm13040960

**Published:** 2024-02-07

**Authors:** Pablo Palacios, Isabel Palacios, Ana Palacios, Juan Carlos Gutiérrez, Gonzalo Mariscal, Alejandro Lorente

**Affiliations:** 1 Department of Traumatology and Orthopaedic Surgery, University Hospital HM Sanchinarro, 28050 Madrid, Spain; 2 Institute for Research on Musculoskeletal Disorders, Valencia Catholic University, 46001 Valencia, Spain; 3 Department of Traumatology and Orthopaedic Surgery, University Hospital Ramón y Cajal, 28034 Madrid, Spain

**Keywords:** XLIF, extreme lateral interbody fusion, spine surgery, minimally invasive, meta-analysis

## Abstract

(1) **Objectives:** The objective of this study was to quantify the exact clinical-radiological efficacy and safety of the extreme lateral interbody fusion (XLIF) technique in spinal surgery; (2) **Methods:** A meta-analysis was performed using PubMed, Embase, Scopus, and Cochrane Collaboration Library. Studies focusing on patients surgically treated with XLIF were included. The outcomes were as follows: visual analog scale (VAS) and Oswestry disability index (ODI), radiological outcomes, and adverse events. Cohort studies and case series were also included. Clinical outcomes were assessed at 12 months of age. Data were combined using Review Manager 5.4 and WebPlotDigitizer 13.1.4; (3) **Results:** Nineteen studies with a pool of 1409 patients were included in this meta-analysis. Leg pain VAS and back pain VAS significantly improved at 12 months (SMD 2.75, 95% CI 0.59–4.90; SMD 4.54, 95% CI 1.39–7.69). ODI showed significant improvement (MD 32.51, 95% CI 24.01–41.00) at 12 months. Disc height increased significantly (SMD −2.73, 95% CI −3.58 to −1.88). Lumbar lordosis and segmental lordosis were significantly corrected postoperatively (MD −2.44, 95% CI −3.45 to −1.43; MD −2.55, 95% CI −3.61 to −1.48). The fusion rates at 12 months ranged from 85.0% to 93.3%. The most frequent complications were transient neurological conditions (2.2%), hardware failure (1.9%), and transient pain (1.8%). The most frequent serious complications were nerve root injury (1.0%), gastrointestinal impairment (0.7%), and vertebral fractures (0.6%); (4) **Conclusions:** This is the first meta-analysis of the specific use of XLIF in spinal surgery. This study demonstrates that the XLIF technique in spine surgery is associated with good clinical and radiological results and a low complication rate.

## 1. Introduction

Low back pain and degenerative conditions of the lumbar spine are widespread health issues affecting millions of people worldwide. It has been estimated that over 80% of the population develops signs of lumbar disc degeneration by the age of 60 [1]. Spondylolisthesis has a prevalence ranging between 3–4%, increasing the risk of low back pain and radiculopathy [2].

Lumbar spinal stenosis is also a frequent cause of nerve root impingement and neurogenic claudication in older adults, with a community prevalence of up to 39% [3]. The personal and economic burden of these degenerative lumbar conditions is massive, estimated to account for billions of dollars annually in health care costs and lost productivity in Western nations alone [4].

XLIF or extreme lateral interbody fusion is a technique in spine surgery that uses a lateral approach, avoiding the risk of vascular and peritoneal injury of the anterior approach (ALIF) and avoiding injury to the musculature or facet injuries by more posterior approaches, such as PLIF or TLIF. Although several approaches have been described, the best remains debatable [5]. XLIF is one of the most minimally invasive techniques that reduces hospital stay and iatrogenic complications. XLIF has also been associated with faster recovery, pain relief, greater functionality, and shorter hospital stay compared to conventional open surgery [6,7].

XLIF has been used for a wide variety of pathologies including degenerative scoliosis, spondylolisthesis, lumbar canal stenosis, and degenerative disc disease. It avoids anterior or posterior ligament resection and increases the height of the disc space. Most studies tend to be case series with variable results in terms of exact improvement in pain, quality of life, and functionality. However, some drawbacks of the XLIF technique include controversy regarding the frequency of associated complications [8]. This is due to its transpsoas approach, which presents risks to nervous structures such as the lumbar plexus, ilioinguinal, iliohypogastric, and genitofemoral nerves [9]. The frequency of complications also depends on many factors, including location; for example, the L4-L5 segment presents a greater risk of associated complications because of its close relationship with the lumbar plexus [10,11].

Individual studies reported different findings regarding XLIF outcomes. Some studies found lower intraoperative blood loss and satisfactory radiographic corrections with XLIF than with other techniques [12]. Others specified that XLIF may be more suitable for certain anatomical features, such as elevated psoas major muscle, psoas major hypertrophy, or high iliac crest [13]. Additional comparative analyses have shown that XLIF may be associated with fewer complications than direct decompression techniques such as TLIF [14]. Further studies found that XLIF and OLIF can restore sagittal alignment [15]. However, a higher rate of nonunion and neurological complications was reported in spondylolisthesis patients treated with XLIF [16]. Some authors did not recommend the use of XLIF for L5-S1 fusion due to anatomical complexity [17], although its minimally invasive approach has led to its use as a treatment of choice in elderly patients with comorbidities [18].

Given the variation in the outcomes reported by different authors using the XLIF technique, a meta-analysis was proposed to provide the best evidence to clarify and quantify the exact improvement of XLIF clinically and radiologically, as well as to determine, with the best evidence, the frequency of complications associated with this technique.

## 2. Materials and Methods

### 2.1. Eligibility Criteria

**Figure 1:**
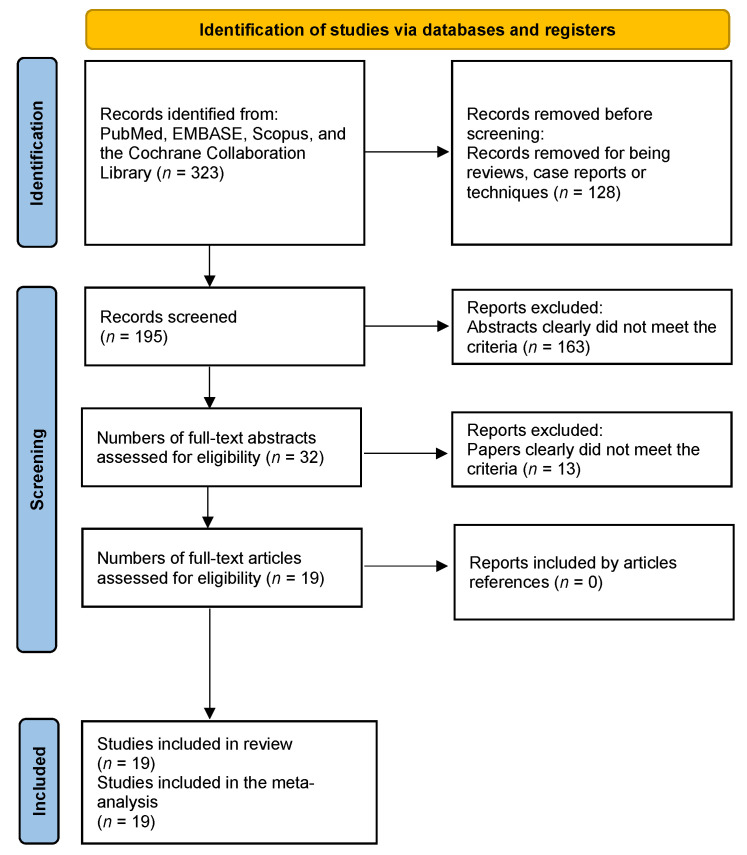
Study selection flow diagram (Preferred Reporting Items for Systematic reviews and Meta-Analysis).

This study had a written protocol with review questions, search strategy, inclusion/exclusion criteria, and risk of bias assessment (PROSPERO: CRD42023398883). It followed PRISMA guidelines (Figure 1) [19], and the language was limited to English. There were no restrictions regarding the year of publication. The research question was conducted following the PICOS strategy: (P) patients with spinal pathology were treated surgically with XLIF (spinal pathology was considered as follows: adult spinal deformity, spondylolisthesis, spinal stenosis, disc pathology, and infection); (I) the intervention was the XLIF technique; (C) this was a meta-analysis of single-arm or serial studies, so there was no comparison (the comparison was considered the post-intervention); (O) the outcomes were XLIF efficacy assessed by scores on the functional, pain, or quality-of-life scales, as well as the radiological outcomes, generally assessed by fusion rate and coronal and sagittal measures; and (regard) adverse events offered by the studies (S)—we included case series or cohort studies (prospective or retrospective cohort studies). When more than one technique was assessed, only the XLIF arm was considered. The diagnosis of spinal pathology was made clinically and by imaging (radiography, MRI, and/or CT). We excluded patients younger than 16 years, with disabling systemic disease, follow-up less than 6 months, previous surgeries, duplicate data, incomplete data, or non-shared variables.

### 2.2. Information Sources

A systematic search of the literature using PubMed, EMBASE, Scopus, and the Cochrane Collaboration Library databases was carried out. Language was limited to English. There was no restriction on the year of publication. Only published studies have been conducted to date.

### 2.3. Search Methods for Identification of Studies

Two reviewers independently agreed on the selection of eligible studies and reached a consensus regarding which studies to include. An initial screening of titles and abstracts was performed to eliminate studies outside the scope of the review. In case of uncertainty based on title or abstract, the full text of each article was examined for further evaluation. If a consensus could not be reached, a third review author was asked to complete the data extraction form and discuss the article with the other two authors until a consensus was reached. All disagreements were resolved by discussion. We consulted experts to assess which variables would be of most interest, as well as to evaluate the shortcomings of previous studies.

### 2.4. Data Extraction and Data Items

Two authors independently reviewed the studies for data extraction. If there was a conflict, a third reviewer participated in data extraction to resolve it. Baseline characteristics, clinical and functional variables, minimal clinically relevant differences, radiological findings, and adverse events were extracted from the studies. The baseline data included the study, region of publication, period of publication, device, number of patients, diagnosis method, type of study, surgeon experience, age, sex, fusion support, number of fused segments, and BMI. We also recorded the length of hospital stay (LOS), blood loss, and time of surgery (OR time). The clinical variables were VAS leg pain, VAS back pain, and Oswestry disability index (ODI). Clinical variables were assessed preoperatively and one year after the procedure. The radiological outcomes included fusion rate, disc height, lumbar lordosis L1–L5, and segmental lordosis. Radiological variables were assessed pre-and postoperatively. Fusion was defined as a bridge between the trabecular interbody bone and at least two consecutive CT slices. In cases where the follow-up of any study did not exactly match that of the majority of studies, the closest follow-up was approximated. Missed data were estimated using Cochrane calculators, review manager, web plot digitizer, or estimates recommended in the Cochrane book. The minimal clinically important difference (MCID) was included in the results, based on previous studies that analyzed these scales. The MCID for VAS and ODI were 5.2 and 12.8 points, respectively [20,21]. We then assessed whether MCID was achieved (Yes/No).

### 2.5. Assessment of Risk of Bias in Included Studies

The quality of the included studies was assessed independently by two authors using the methodological index for non-randomized studies (MINORS) criteria [22]. The maximum score was 24 for the comparative studies and 16 for the non-comparative studies. For non-comparative studies, scores of 0–4 corresponded to very low quality, 5–7 corresponded to low quality, 8–12 corresponded to fair quality, and ≥13 corresponded to high quality, respectively. For comparative studies, scores of 0–6 corresponded to very low quality, 7–10 corresponded to low quality, 11–15 corresponded to fair quality, and ≥16 corresponded to high quality, respectively.

### 2.6. Assessment of Results

Meta-analysis was performed using the Review Manager 5.4 software package provided by the Cochrane Collaboration. For dichotomous variables, odds ratios (ORs) with a 95% confidence interval (CI) were calculated. The mean difference (MD) and 95% CI were calculated for the continuous variables. The standard mean difference (SMD) was calculated for continuous variables that did not share the same measurement units. Heterogeneity was checked using both the chi^2^ and I^2^ tests. I^2^ varied from 0 to 100%, considering the values of 25, 50%, and 75% as low, moderate, and high heterogeneity, respectively. A fixed-effects model was adopted if there was no statistical evidence of heterogeneity, and a random-effects model was adopted if significant heterogeneity was observed. WebPlotDigitizer version 13.1.4 was used to obtain accurate information from the figures in the articles.

### 2.7. Risk of Bias across the Studies

We assessed the possibility of publication bias by evaluating a funnel plot (Review Manager 5.4 software package provided by the Cochrane Collaboration) of the trial mean differences for asymmetry, which can result from non-publication of small trials with negative results. We acknowledge that other factors, such as differences in trial quality or true study heterogeneity, could produce asymmetry in funnel plots.

### 2.8. Additional Analyses

Subgroup analyses were conducted based on the following two key factors: whether the XLIF procedure was performed as a stand-alone technique or with posterior stabilization and whether it was a single-level or multilevel XLIF procedure.

A sensitivity analysis was also carried out using the Review Manager 5.4 software package, eliminating the top-weight study from the comparisons in all outcomes. The sensitivity analysis evaluates the robustness and certainty of the conclusions against modifications in the data and methods.

## 3. Results

### 3.1. Study Selection

A total of 19 studies were identified for inclusion in the meta-analysis [7,12,13,14,15,16,17,18,23,24,25,26,27,28,29,30,31,32,33]. The searches in PubMed, EMBASE, Scopus, and the Cochrane Collaboration Library provided a total of 323 citations. Of these, 128 studies were excluded as case reports, techniques, or reviews. Of these, 163 studies were discarded because, after reviewing the abstracts, it appeared that these papers clearly did not meet the criteria. The full texts of the remaining 32 citations were examined in more detail. Thirteen studies did not meet our inclusion criteria. After adjusting for duplicates, 19 studies met the inclusion criteria and were included in the systematic review and meta-analysis (Figure 1).

### 3.2. Study Characteristics

Table 1 presents the baseline characteristics of the included studies. Nineteen studies were included, with a total of 1409 patients. The mean age ranged from 51.0 to 71.1 years. The proportion of women ranged from 40.3% to 87.6%. These studies were published between 2010 and 2022. One study was a prospective cohort, 11 were retrospective cohort studies, and seven case series. Most surgeons have experienced this procedure. The use of two or more levels ranged from 10% to 83.3%. The methods employed individually in each study for cage implantation, fusion technique, and biomaterials used are given in Supplementary Table S1.

### 3.3. Risk of Bias

The risk of bias assessment results is presented in Table 2. All studies—cohort studies and clinical series—showed at least fair quality. Most studies were of high quality.

### 3.4. Outcomes

Table 3 shows the results for the LOS, BL, and OR time variables. LOS was reported in three studies. The mean LOS was 12.3 and ranged from 3.6 to 25.8 days. However, when patients with infection were excluded, the mean LOS was 5.6, with ranges from 3.6 to 7.5 days. The mean blood loss was 180.0 mL varying between 49.2 Â mL (minimum, and 528.0 mL, (maximum). The mean OR time was 182.9 min, ranging from 85.0 min, minimum, to 347.5 min, maximum).

**Figure 2:**
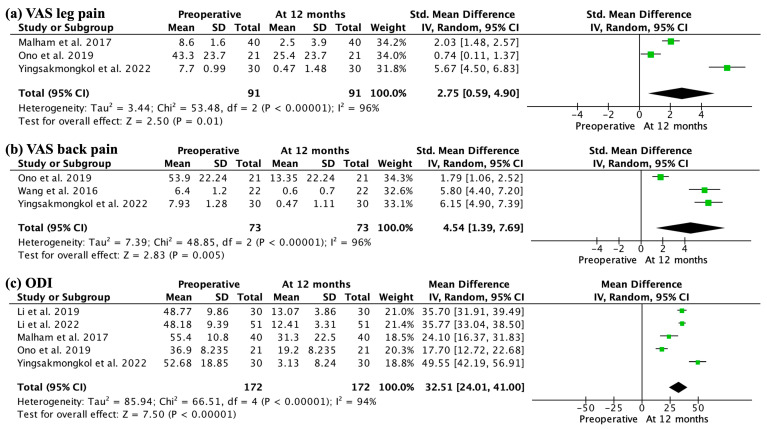
Forest plot showing significant improvement at 12 months of the pain and functional outcomes. VAS leg pain (**a**), VAS back pain (**b**), and ODI (**c**) also demonstrate a relevant clinical improvement.

VAS leg pain and back pain improved significantly at the 1-year follow-up (SMD 2.75, 95% CI 0.59–4.90; participants = 182; studies = 3; I2 = 96% and SMD 4.54, 95% CI 1.39–7.69; participants = 146; studies = 3; I2 = 96%, respectively) (Figure 2a,b). ODI showed significant improvement at the 1-year follow-up (MD 32.51, 95% CI 24.01–41.00; participants = 344; studies = 5; I2 = 94%) (Figure 2c). VAS and ODI scores exceeded the MCID.

**Figure 3:**
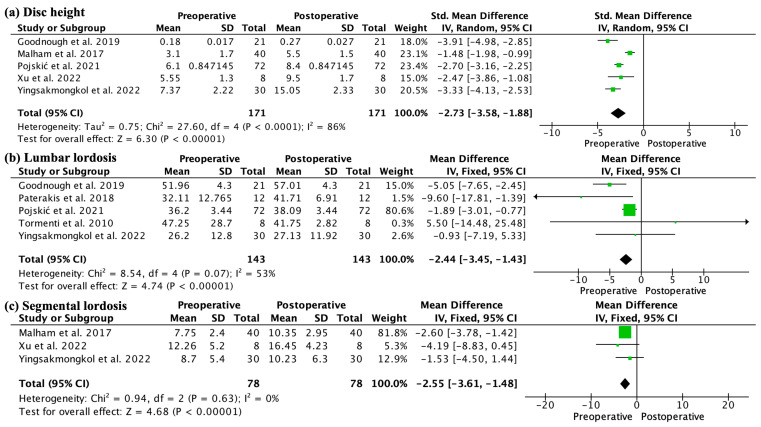
(**a**) Forest plot showing the significant increase in disc height postoperatively (*p* < 0.001); (**b**,**c**) Forest plot showing a significant correction of lumbar lordosis and segmental lordosis postoperatively (*p* < 0.001).

The disc height increased significantly postoperatively (SMD −2.73, 95% CI −3.58 −1.88; participants = 342; studies = 5; I2 = 86%) (Figure 3a). Lumbar lordosis and segmental lordosis were corrected significantly after surgery (MD −2.44, 95% CI −3.45, −1.43; participants = 286; studies = 5; I2 = 53% and MD −2.55, 95% CI −3.61 −1.48; participants = 156; studies = 3; I2 = 0%, respectively) (Figure 3b,c). At 6 months, the fusion rates ranged from 36.1% to 63.3%, at 9 months from 53.5% to 58.0%, at 12 months from 85.0% to 93.3% (Table 4).

Complications were reported in 17 studies with a total of 1247 patients and are listed in Table 5. The most frequent mild complications were transient neurological conditions (2.2%), hardware failure (1.9%), and transient pain (1.8%). The most frequent serious complications were nerve root injury (1.0%), gastrointestinal impairment (0.7%), and vertebral fractures (0.6%).

### 3.5. Additional Analyses

Regarding the subgroup analyses, the Oswestry disability index (ODI) showed no significant differences between stand-alone XLIF (MD 30.41, 95% CI 19.09 to 41.73; participants = 140; studies = 5; I2 = 86%) and posterior stabilization XLIF (MD 34.14, 95% CI 19.17 to 49.10; participants = 204; studies = 5; I2 = 97%). However, single-level XLIF demonstrated significantly worse ODI outcomes (MD 36.48, 95% CI 24.59 to 48.36; participants = 200; studies = 5; I2 = 91%) than multi-level XLIF (MD 17.70, 95% CI 12.72 to 22.68; participants = 42; studies = 5; I2 = 0%).

In terms of radiological outcomes, there were no differences in disc height between posterior stabilization XLIF (SMD −3.19, 95% CI −3.88 to −2.50; participants = 246; studies = 5; I2 = 61%) and stand-alone XLIF (SMD −1.75, 95% CI −2.61 to −0.89; participants = 96; studies = 5; I2 = 42%). Similarly, single-level XLIF (SMD −2.79, 95% CI −3.76 to −1.81; participants = 326; studies = 5; I2 = 89%) and multi-level XLIF (SMD −2.47, 95% CI −3.86 to −1.08; participants = 16; studies = 5; I2 = 0%) showed no significant differences.

There were no differences in lumbar lordosis between posterior stabilization XLIF (MD −2.33, 95% CI −3.34 to −1.31; participants = 262; studies = 5; I2 = 46%) and stand-alone XLIF (MD −9.60, 95% CI −17.81 to −1.39; participants = 24; studies = 5; I2 = 100%). Single-level XLIF (MD −2.46, 95% CI −3.47 to −1.45; participants = 270; studies = 5; I2 = 62%) also showed no differences compared to multi-level XLIF (MD 5.50, 95% CI −14.48 to 25.48; participants = 16; studies = 5; I2 = 0%).

**Figure 4:**
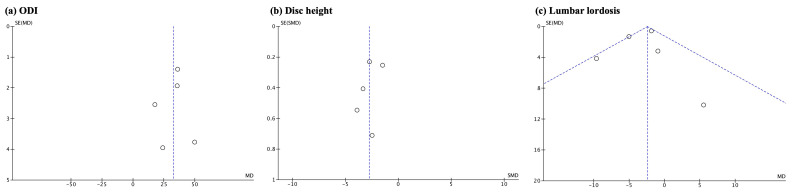
Funnel plot displaying the possibility of publication bias due to the observed asymmetry. (**a**) ODI; (**b**) disc height; (**c**) lumbar lordosis.

Visual inspection of the funnel plot revealed asymmetry, indicating the possibility of publication bias (Figure 4).

A sensitivity analysis was performed by eliminating the top-weight studies from the comparisons of all outcomes. None of the variables examined changed the direction of the results. 

## 4. Discussion

This meta-analysis quantified the efficacy and safety of XLIF for spinal surgery. XLIF significantly improved pain and function at 1-year follow-up. In addition, the MCID was exceeded in all cases. Radiological parameters (disc height, fusion rate, and lumbar and segmental lordosis) were significantly corrected. The most frequent complication was a transient neurological condition. The quality of the studies was generally fair or high.

Minimally invasive surgery is the current trend because of less damage to paraspinal musculature and early recovery [6,7]. However, the duration of hospitalization could be overestimated by studies that included infections due to prolonged antibiotic treatment regimens. It is crucial to consider the anatomy and note that XLIF is limited superiorly by the axilla and inferiorly by the iliac crest [34]. Better visualization was described with XLIF, especially in infectious processes, because of the great exposure of bodies and discs.

In this study, it was not possible to compare the coronal angles, although it was demonstrated that the most important issue is to re-establish lordosis and achieve fusion [35]. Sagittal balance in patients with adult spinal deformity is crucial to avoid pain and improve quality of life [36]. Sagittal balance is the best predictor of quality of life [37]; however, in some cases, because of patient age and bone quality, it is difficult to achieve satisfactory correlations. Studies with a greater number of fused segments [17,18,29] reported lower corrections of radiological parameters (lumbar lordosis and segmental lordosis). They also presented lower scores in the quality of life and functionality scales [17,18,29]. In addition, XLIF achieves significant disc height restoration, which can decrease nerve root compression [38]. In contrast, the addition of fusion to the degenerative spine increases the success rate of reintervention [39]. In this study, the mean fusion rate increased from 48.5% at 6 months, 55.8% at 9 months, and 89.5% at 1 year, although some factors, such as BMP, increased the fusion rate [40]. The studies included in the meta-analysis used BMP in many cases, although allografts, demineralized bone matrices, and β-TCP granules were also used.

The frequency of complications was low, with transient neurological conditions being the most frequent. Complications that required surgical management were related to hardware failure (in up to 25% of cases). Special care should be taken with the L5-S1 lumbar plexus as this injury is one of the greatest concerns [11]. The nerves at greatest risk during surgery are the ilioinguinal, iliohypogastric, lateral femoral cutaneous, and genitofemoral. These sensory nerves cannot be monitored in real-time during surgery. However, these complications can also be attributed to the learning curves. Only 8 of the 19 studies specifically reported the use of intraoperative electromyographic neuromonitoring. The main purpose of utilizing neuromonitoring is to prevent the most common complications of XLIF and transient neurological deficits [41]. Neuromonitoring systems provide real-time, surgeon-directed electrical responses to indicate the proximity of the motor nerves during instrumentation. This helps reduce nerve injury risk by enabling more posterior implant docking, improving clinical outcomes, and decreasing neurological complications [32]. Goodnough et al. used neuromonitoring for cage insertion and screw placement [16]. Tessitore et al. conducted continuous triggered EMG neuromonitoring on dilators [24]. However, most studies that employed neuromonitoring only specified its use and failed to define what constituted a “positive” reading or potential nerve damage. Studies did also not report whether positive readings occurred or how surgeons proceeded in such cases. The lack of standardized neuromonitoring protocols limits the ability to determine their true impact. Future research should describe the monitoring methodology, thresholds for positive responses, and intraoperative management based on readings in detail. Experience level was only reported in a few studies, although mentorship is crucial for early surgical cases. Strict adherence to techniques, including neuromonitoring protocols, also remains vital for maximizing safety.

### Limitations and Strengths

This study had several limitations. In many cases, Cochrane missing data calculators were used, along with the procedures described in the Cochrane book. Most studies were cohort studies or retrospective clinical series; therefore, the level of evidence was low. Regarding the characteristics of the cages used, their size could not be purchased. In some cases, disc height was measured in the anterior and posterior regions. The method and modality used to determine fusion were explained in some articles, while others did not explain them in detail. In addition, the standard mean difference was used for the VAS variables because one study used different values for the scales. In addition, the 1-year follow-up period was relatively short to assess the efficacy of the surgical technique. However, this is an important limitation to highlight for future studies, which should aim for long-term follow-up. A limitation of the studies included in this review is that they did not provide complete details regarding the meaning and results of electromyographic neuromonitoring used during the surgical procedures assessed. This is especially relevant, given that nerve complications are one of the most common adverse events associated with the XLIF surgical technique. Specifically, they did not specify the purpose of using this monitoring technique or what readings were considered ‘positive.’ They also did not report measures taken when these types of abnormal readings were detected. The lack of this methodological information makes it difficult to understand the actual value of the neuromonitoring provided by the procedures. Another limitation of the present study is the impossibility of determining the precise indications for which the XLIF procedures were performed as different etiologies were included. Future studies should focus on specific pathologies or adjust the results for each etiology. Finally, the variety of etiologies included in the meta-analysis should be considered; subgroups could not be made to control for the etiological factor because the number of articles in each group would be insufficient to draw solid conclusions.

## 5. Conclusions

The use of XLIF in spinal surgery resulted in significant pain relief after 1 year. Moreover, this improvement is clinically relevant. XLIF also significantly improved functionality after one year, and this change was clinically relevant. Disc height was significantly restored, but whether this increase improves nerve compression or if decompression of the canal is required is unknown. The lumbar and segmental lordosis improved significantly. Future studies should analyze the correlation between radiological parameters and quality of life or functionality, specifically for the XLIF technique. The fusion rate at 1 year was satisfactory (between 85.0% and 93.3%). Finally, the complication rate was relatively low and serious adverse events were infrequent. Transient neurologic conditions (2.2%) and hardware failure (1.9%) were the most frequent adverse events, with the latter requiring reoperation in 25% of cases. Future studies should describe more precisely the meaning and management of the results obtained by intraoperative electromyographic neuromonitoring, especially considering the high incidence of nerve complications associated with the XLIF technique.

## Figures and Tables

**Table 1:** Baseline characteristics of the 19 included studies.

Study	Region	Period	*n*	Type of Study	Surgeon	Age	% Female	Fusion ≥ 2 Segments	BMI
Goodnough et al., 2019 [7]	USA	2008 to 2012	21	Retrospective cohort	Fellows	65.7	66.6%	26.3%	28.6
Hiyama et al., 2020 [14]	Japan	2016 to 2019	62	Retrospective cohort	-	70.2	40.3%	30.6%	-
Khajavi et al., 2015 [28]	USA	2008 to 2012	187	Retrospective cohort	-	61.0	66.0%	-	-
Li et al., 2019 [27]	China	-	30	Retrospective cohort	-	58.4	60.0%	20.0%%	-
Li et al., 2022 [13]	China	2017 to 2019	51	Retrospective cohort	-	56.7	62.7%	-	-
Malham et al., 2012 [32]	Australia	2011	30	Case series	-	62.7	66.7%	-	26.7
Malham et al., 2017 [25]	Australia	2011 to 2012	40	Retrospective cohort	Expert	63.5	70.0%	32.5%	26.9
Ono et al., 2019 [29]	Japan	2014 to 2017	21	Retrospective cohort	-	71.1	81.0%	71.4%	-
Paterakis et al., 2018 [18]	Greece	2008 to 2017	12	Case series	-	64.5	100.0%	83.3%	-
Pojskić et al., 2021 [16]	Germany	2010 to 2018	72	Case series	-	66.6	44.4%	33.3%	-
Rodgers et al., 2011 [31]	USA	Since 2006	600	Retrospective cohort	Senior	61.4	62.0%	-	31.1
Schonauer et al., 2017 [23]	Switzerland	2014 to 2016	41	Retrospective cohort	-	67.4	45.7%	14.3%	27.6
Tessitore et al., 2016 [24]	Switzerland	2014 to 2015	20	Case series	-	67.4	50.0%	10.0%	27.7
Timothy et al., 2019 [26]	UK	2008 to 2011	14	Case series	-	51.0	NA	14.3%	-
Tohmeh et al., 2014 [33]	USA	2008 to 2012	140	Prospective cohort	-	60.7	44.6%	-	29.1
Tormenti et al., 2010 [12]	USA	2007 to 2009	8	Retrospective cohort	-	60.0	-	-	-
Wang et al., 2016 [30]	China	2012 to 2014	22	Case series	Experienced	-	45.5%	-	-
Xu et al., 2022 [17]	China	-	8	Case series	Experienced	60.4	87.6%	50.0%	-
Yingsakmongkol et al., 2022 [15]	Thailand	2016 to 2019	30	Retrospective cohort	Senior	63.5	73.3%	-	-

-: Missing data.

**Table 2:** Assessment of the quality of studies through the methodological index for non-randomized studies (MINORS).

Study	Clearly Stated Aim	Consecutive Patients	Prospective Collection Data	Endpoints	Assessment Endpoint	Follow-Up Period	Loss Less than 5%	Study Size	Adequate Control Group	Contemporary Group	Baseline Control	Statistical Analyses	Minors
Goodnough et al., 2019 [7]	2	2	0	2	2	2	1	2	2	2	2	2	21
Hiyama et al., 2020 [14]	2	2	2	2	2	2	1	2	2	2	2	2	23
Khajavi et al., 2015 [28]	1	2	2	2	2	2	1	2	2	2	2	2	22
Li et al., 2019 [27]	2	1	0	2	2	2	2	2	2	2	2	2	21
Li et al., 2022 [13]	1	2	0	2	2	2	1	2	2	2	2	2	20
Malham et al., 2012 [32]	2	2	2	2	2	2	1	2	-	-	-	-	15
Malham et al., 2017 [25]	2	2	1	2	2	2	1	1	2	2	2	2	21
Ono et al., 2019 [29]	2	2	0	2	2	2	1	1	2	2	2	2	20
Paterakis et al., 2018 [18]	1	2	1	2	2	2	1	1	-	-	-	-	12
Pojskić et al., 2021 [16]	2	2	0	2	2	2	2	2	-	-	-	-	14
Rodgers et al., 2011 [31]	1	2	1	1	1	2	1	2	-	-	-	-	11
Schonauer et al., 2017 [23]	1	2	0	2	2	2	1	2	2	2	2	2	20
Tessitore et al., 2016 [24]	1	2	0	2	2	2	1	1	-	-	-	-	11
Timothy et al., 2019 [26]	2	2	0	2	2	2	2	1	-	-	-	-	13
Tohmeh et al., 2014 [33]	2	2	0	2	2	2	0	2	1	2	1	2	18
Tormenti et al., 2010 [12]	1	2	1	2	2	2	2	1	-	-	-	-	13
Wang et al., 2016 [30]	1	2	0	2	2	1	2	1	-	-	-	-	11
Xu et al., 2022 [17]	1	1	1	2	2	2	1	0	-	-	-	-	10
Yingsakmongkol et al., 2022 [15]	2	2	1	2	2	2	1	2	2	2	2	2	22

**Table 3:** Length of stay (LOS), blood loss (BL), and operating (OR) time.

Study	LOS (Days)	BL (mL)	OR Time (min)
Hiyama et al., 2020 [14]	-	84.4	109.9
Li et al., 2019 [27]	-	122.7	106.2
Li et al., 2022 [13]	-	63.7	85.0
Paterakis et al., 2018 [18]	-	102.0	118.0
Schonauer et al., 2017 [23]	-	528.0	241.0
Wang et al., 2016 [30]	25.8	249.8	347.5
Xu et al., 2022 [17]	7.5	240.0	311.4
Yingsakmongkol et al., 2022 [15]	3.6	49.17	144.0

-: Missing data.

**Table 4:** Fusion rate achieved at 6, 9, and 12 months.

Fusion Rate	6 Months	9 Months	12 Months
Li et al., 2019 [27]	63.3%	-	93.3%
Li et al., 2022 [13]	-	-	92.2%
Malham et al., 2012 [32]	46.0%	58.0%	85.0%
Malham et al., 2017 [25]	36.1%	53.5%	87.6%

-: Missing data.

**Table 5:** Complications.

Complications	*n*	%
Retroperitoneal hematoma	1	0.08
Hardware failure *	24	1.92
Wound problems	14	1.12
Adjacent segment disease	17	1.36
Numbness	1	0.08
Transient hypoesthesia	3	0.24
Permanent hypoesthesia	1	0.08
Transient pain	23	1.84
Transient neurologic conditions	27	2.17
Pleural tear	3	0.24
Dural tear	6	0.48
Nerve root injury **	12	0.96
Gastrointestinal impairment †	9	0.72
Vertebral infection	2	0.16
Endplate injury	4	0.32
Psoas weak	1	0.08
Pneumonia	6	0.48
Infarction	2	0.16
Urinary retention	4	0.32
Urinary incontinence	1	0.08
Anemia requiring transfusion	4	0.32
Vertebral fracture ††	8	0.64
Deep vein thrombosis	1	0.08
Pulmonary embolism	3	0.24
Atrial fibrillation	5	0.40
Peritoneal catheter	1	0.08
Bowel injury	2	0.16
Meningitis	1	0.08
Hemodynamic instability	2	0.16
Cerebrospinal fluid leak	1	0.08

* Cage displacement: 12 (2 underwent surgery); cage breakage: 3 (1 underwent surgery); screw displacement: 5 (2 underwent surgery); screw break: 1 (1 underwent surgery). ** Motor deficit: 1; radiculopathy: 8. † Motor deficit: 1; radiculopathy: 8. †† Five patients underwent surgery.

## Data Availability

No new data were created or analyzed in this study. Data are contained within the article and supplementary materials.

## Supplementary Materials

The following supporting information can be downloaded at: https://www.mdpi.com/article/10.3390/jcm13040960/s1. Reference [42] is cited in the Supplementary Materials.
